# Supplementary material for: Conserved linear dynamics of single-molecule Brownian motion
Source: Nat Commun. 2017 Jun 6;8:15675. doi: 10.1038/ncomms15675 (PMC5467176; doi:10.1038/ncomms15675)
Supplement: Supplementary Information — Supplementary Figures, Supplementary Notes, Supplementary Methods and Supplementary References [file ncomms15675-s1.pdf]

Supplementary Figures

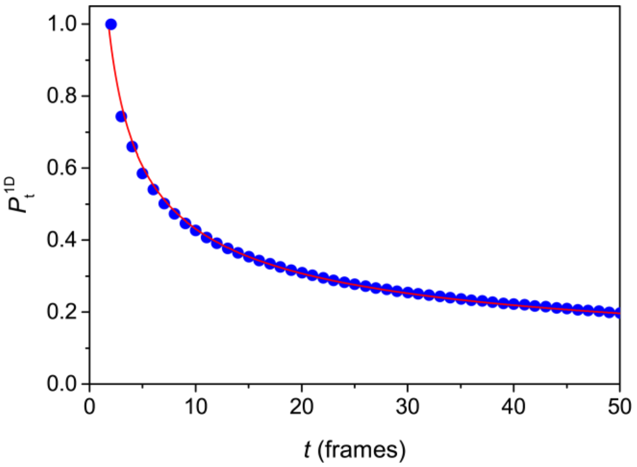

Supplementary Figure 1. Lattice occupancy analysis of 1D random diffusion trajectories. The average probability of occurrence of visits to new lattice sites at time *t* ($\langle P_t^{1D} \rangle$) obtained from 100 simulated 1D random diffusion trajectories. The lattice size (*m*) was set to 160 nm. The step sizes of the trajectories were generated using Eq. 5 (*r* = 160 nm). The red line shows the fitting to Eq. 3.

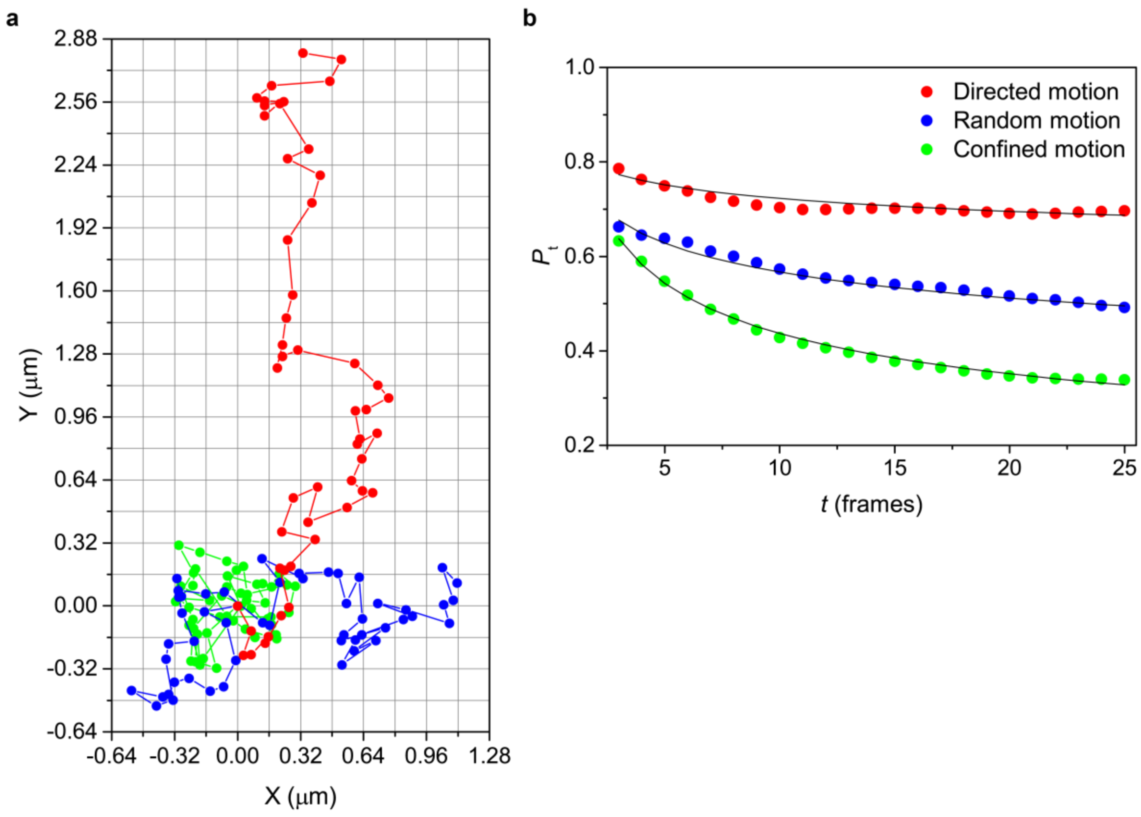

Supplementary Figure 2. Analysis of the directed, random and confined motions by using lattice occupancy analysis. (a) Simulated 2D trajectories (50 frames each) of particles diffusing in directed (red), random (blue) and confined (green) fashions. The step sizes of the 2D random walk were generated by using Eq. 5 (r = 160 nm). The trajectories were mapped onto a 2D square lattice of size m = 160 nm. **(b)** The calculated P_t (Eq. 1) of visits to new lattice sites of the three trajectories shown in **a**. After fitting the data to Eq. 4 (black lines), the calculated scaling exponents (β) for the directed, random and confined motions were −0.05, −0.15 and −0.31, respectively. Note that the directed and confined modes of diffusion show low lattice occupancy mode (high P_{25} value) and high lattice occupancy mode (low P_{25} value), respectively.

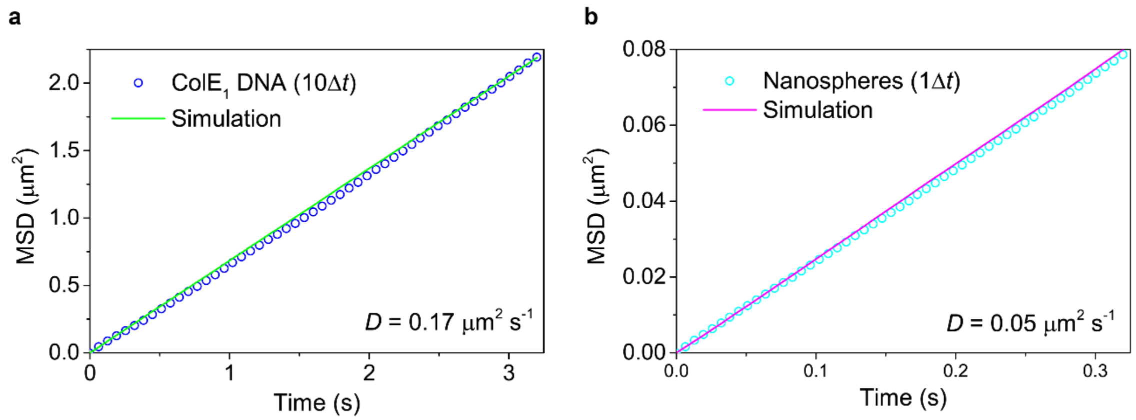

Supplementary Figure 3. Analyses of the diffusion trajectories of ColE$_1$ DNA and nanospheres by using mean squared displacement analysis (MSD). (a) MSD-10Δ*t* profile of ColE$_1$ DNA. The solid line is the MSD-10Δ*t* profile obtained from the S_rA_r simulated trajectory. **(b)** MSD-Δ*t* profile of the nanospheres. The plot shows the averaged MSD-Δ*t* profiles of approximately 50 nanospheres. The solid line is the MSD-1Δ*t* profile obtained from the S_rA_r simulated trajectory.

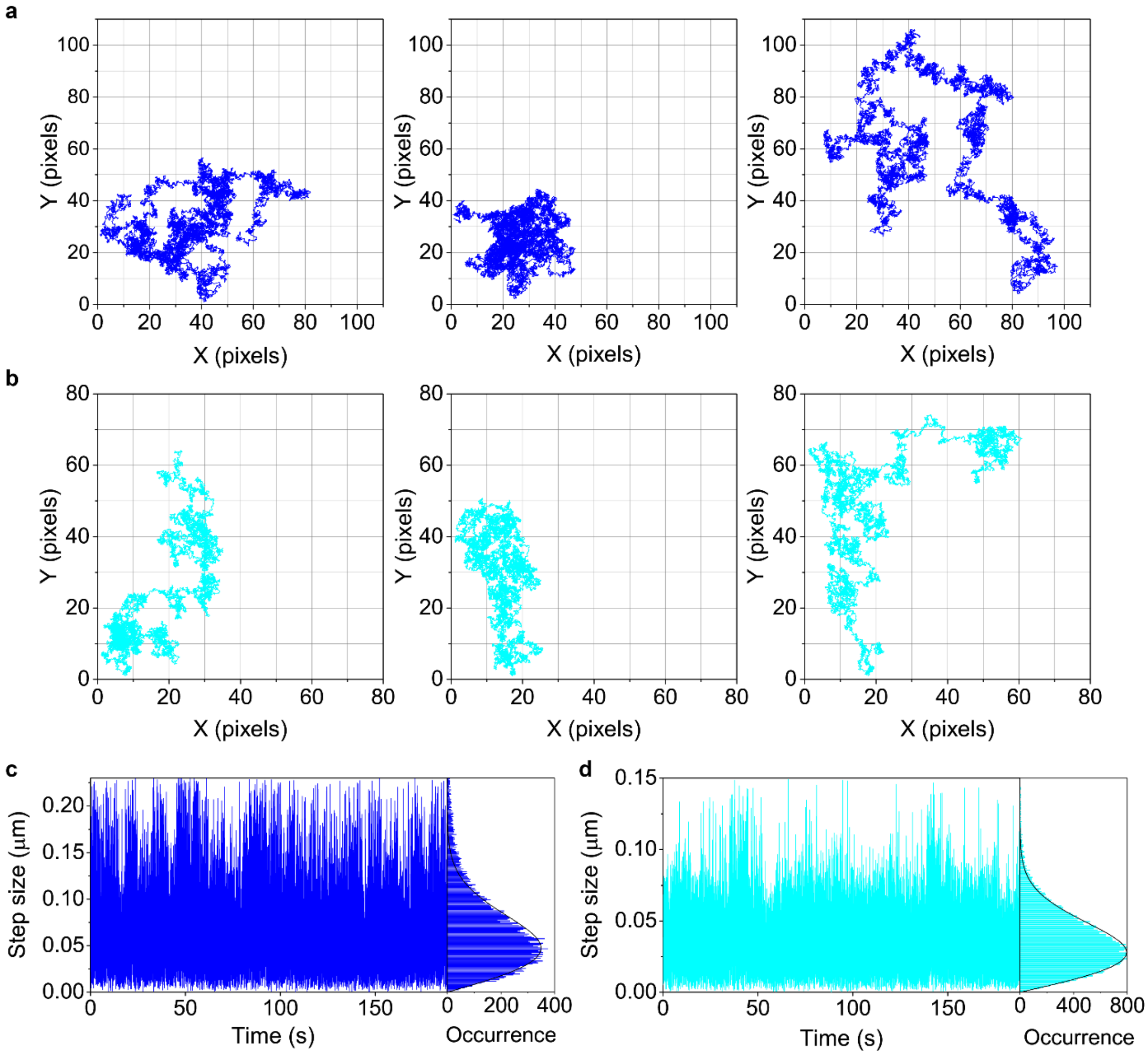

Supplementary Figure 4. End-to-end joined trajectories of ColE$_1$ DNA and nanospheres. **(a)** Three shuffled replicates obtained by randomizing the order of the connections between the original trajectories of the DNA. Pixel size = 0.16 μm. **(b)** Three shuffled replicates obtained by randomizing the order of the connections between the original trajectories of the nanospheres. Pixel size = 0.16 μm. **(c)** Temporal profile of step-sizes of ColE$_1$ DNA after connecting the original trajectories end to end. The frequency histogram of the step sizes is shown in the right panel. The solid line shows the fitting of the frequency histogram to Eq. 5, which indicates that the motion agrees with normal diffusion theory. **(d)** Temporal profile of step sizes of the nansopheres after connecting the original trajectories end to end. The frequency histogram of the step-sizes is shown in the right panel. The solid line shows the fitting of the frequency histogram to Eq. 5.

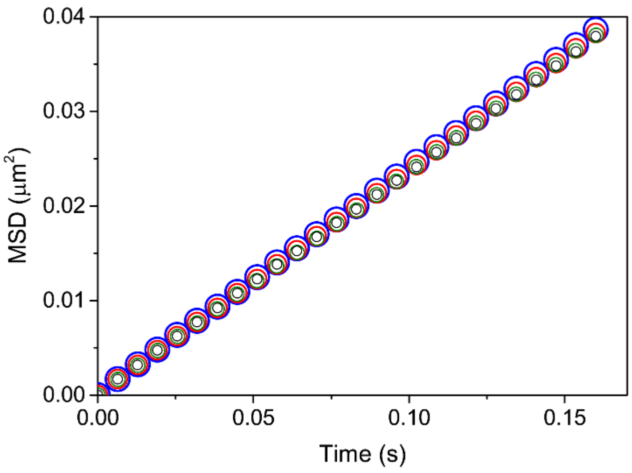

Supplementary Figure 5. MSD analysis of the simulated trajectories of the nanospheres. MSD-Δ*t* profiles of the experimental (blue), the S_rA_r (green), the S_iA_r (red), and the S_rA_i (black) simulated replicates of the nanoparticles.

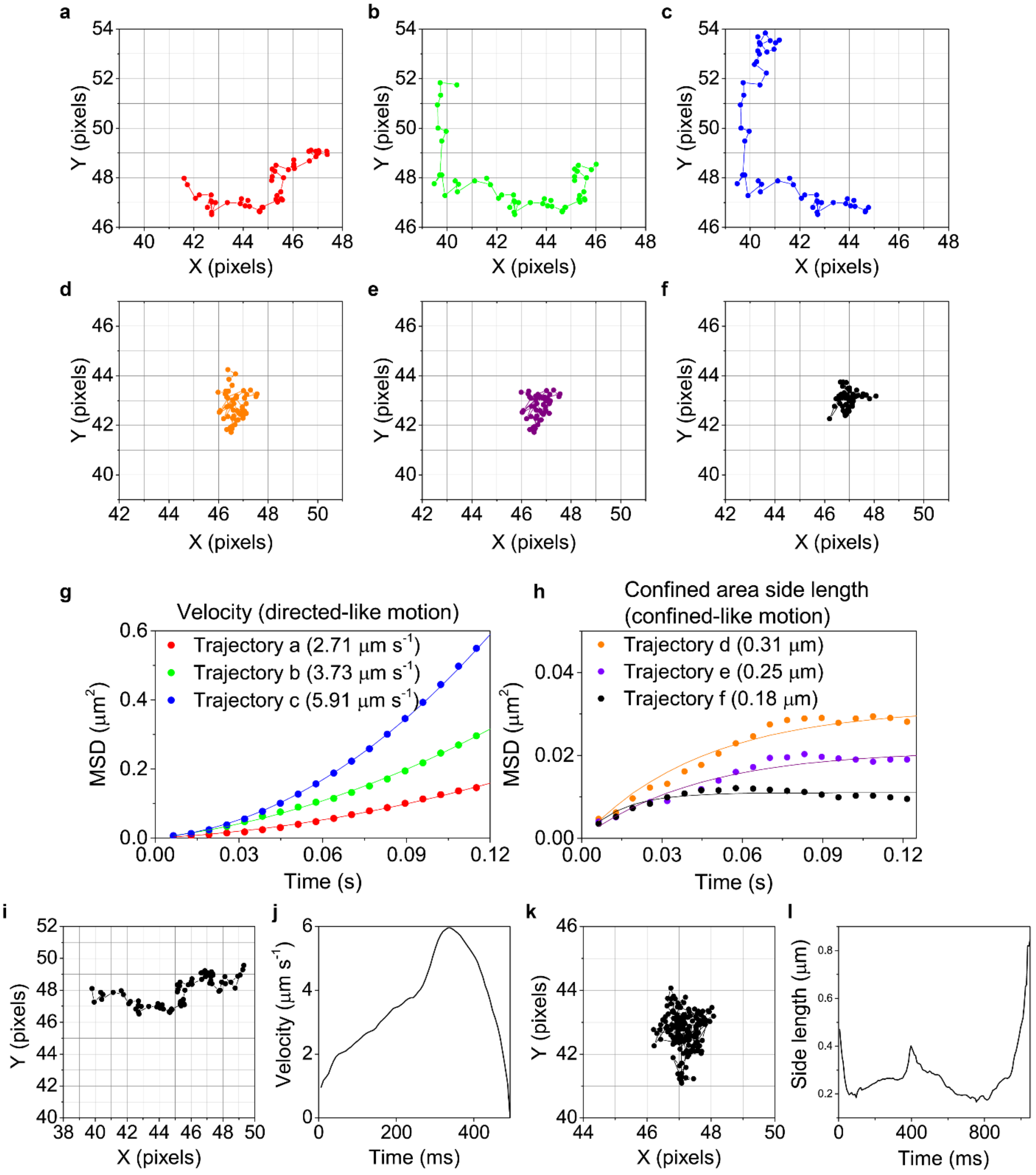

Supplementary Figure 6. Directed-like and confined-like modes of the absolute motion of ColE$_1$ DNA trajectories and the fittings of their MSD-Δ*t* profiles to the confined and directed diffusion models, respectively. (a-c) Three sub-trajectories (50 frames each) of the DNA showing directed-like motion. Pixel size = 0.16 μm. **(d-f)** Three sub-trajectories (50 frames each) of the DNA showing confined-like motion. Pixel size = 0.16 μm. **(g)** MSD-Δ*t* profiles of trajectories **a-c**. The profiles were fitted (solid lines) to Eq. 10 and the velocities of the directed-like motion (*v*) are shown for each profile. **(h)** MSD-Δ*t* profiles of trajectories **d-f**. The profiles were fitted (solid

lines) to Eq. 11 and the side lengths of the confined area (L) are shown for each profile. **(i)** A DNA trajectory showing directed-like motion. **(j)** Temporal change in the velocity of the directed-like mode of motion of the DNA trajectory shown in **(i)**. The plot was obtained by using a sliding window of width 50Δt and by fitting the MSD-Δt profiles to Eq. 10. **(k)** A DNA trajectory showing confined-like motion. **(l)** Temporal change in the side length of the confined area of the DNA trajectory shown in **k**. The plot was obtained by using a sliding window of width 50Δt and by fitting the MSD-Δt profiles to Eq. 11.

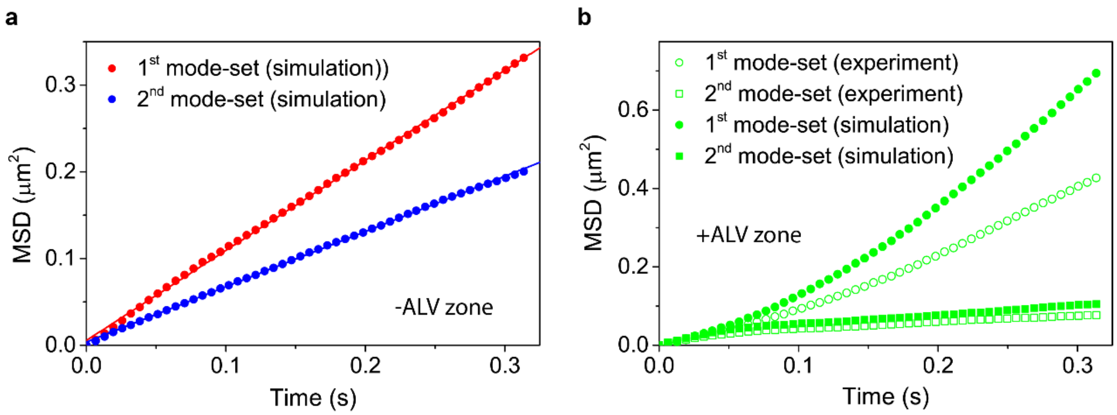

Supplementary Figure 7. MSD analyses of the sub-trajectories obtained by using the threshold values ALVμ_G ± 2.2σ_{ALV}. (a) Averaged MSD-Δ*t* profiles of the sub-trajectories captured in the –ALV zones of the first and second mode-sets of the S_rA_r simulated replicates. The solid lines show linear fitting of the averaged MSD-Δ*t* profiles. The mode of these sub-trajectories agrees with purely random behaviour. **(b)** Averaged MSD-Δ*t* profiles of the sub-trajectories obtained from the +ALV zones of the first and second mode-sets of the experimental and the S_rA_r simulated replicates. Because the +ALV zone of the first mode-set contained information related to high amplitudes of the directed-like mode (see Fig. 5), the captured trajectories showed directed-like motion. Conversely, because the second mode-set contained information related to high amplitudes of the confined-like mode, the captured trajectories showed confined-like motion. The sub-diffusive behaviour (Fig. 2c), which affects the initial slope of the experimental MSD-Δ*t* profiles, caused the experimental profiles to deviate toward lower values when compared with the S_rA_r simulated profiles.

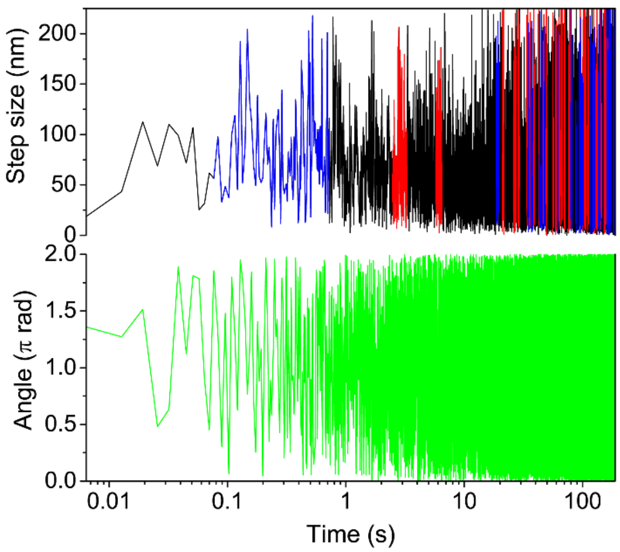

Supplementary Figure 8. The time series of step-sizes and step-directions. Temporal profiles of the step-sizes (top) and the step-directions (bottom). The step-sizes that belong to the c-LO sub-mode are highlighted in red, whereas the step-sizes that belong to the d-HO sub-mode are highlighted in blue.

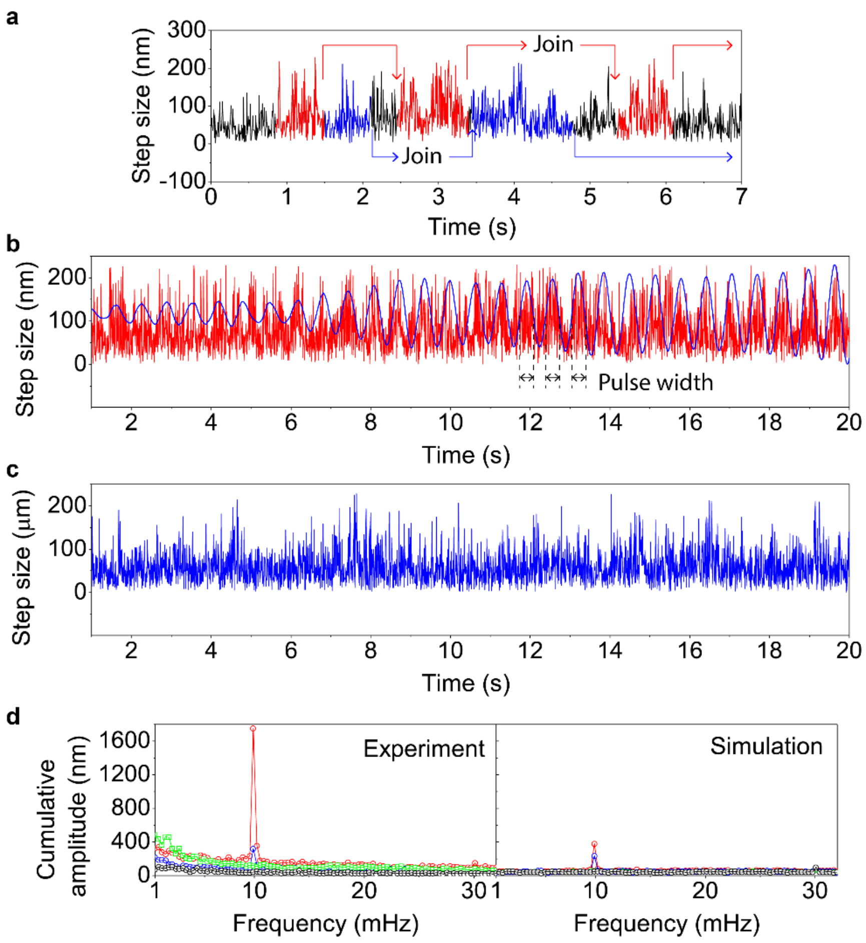

Supplementary Figure 9. Calculation of the characteristic time scale of the c-LO and d-HO sub-modes. (a) Enlarged view of the temporal profile of the step sizes shown in Supplementary Fig. 4c. The step-sizes that belong to the c-LO and d-HO sub-modes are highlighted in red and blue, respectively. The steps of each sub-mode were joined end to end to generate c-LO- and d-HO-joined datasets **b** and **c**. **(b)** c-LO-joined dataset (red) obtained from the temporal profile of the step-sizes shown in Supplementary Fig. 4c. The dataset exhibited a periodic waveform that enabled Fourier transform analyses. The blue signal is the filtered signal obtained after treating the dataset with a band-pass filter (see Supplementary Fig. 10). The characteristic time scale of the c-LO mode ($\tau_{c\text{-}LO}$) is given by the pulse width of the filtered signal. The pulse widths from all of the experimental replicates were averaged to give $\tau_{c\text{-}LO}$ = 0.33 ± 0.16 s. **(c)** d-HO-joined dataset obtained from the temporal profile of the step sizes shown in Supplementary Fig. 4c. The d-HO-joined dataset lacked the periodic waveform essential for the Fourier transform analyses. Therefore, the characteristic time scale cannot be calculated by using the Fourier transform analytical approach. **(d)** Cumulative frequency-magnitude spectra of the c-LO- (red) and d-HO- (blue) joined datasets obtained from the experimental (left) and the simulated S_rA_r trajectories (right). The cumulative frequency-magnitude spectra of the joined datasets obtained from the +ALV zones of the first (green) and the second (black) mode-sets are shown for comparison.

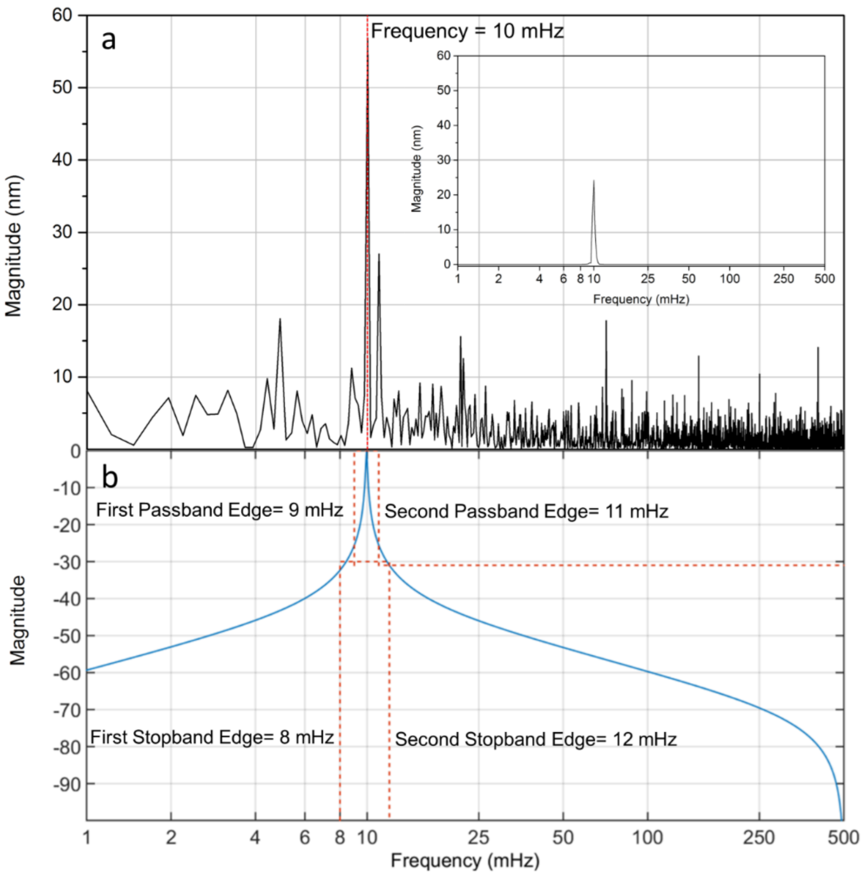

Supplementary Figure 10. Band-pass filtering of the periodic c-LO joined dataset. (a) Frequency magnitude spectrum of the c-LO extended data set of an experimental replicate. The inset is the frequency magnitude spectrum of the c-LO extended data set after treatment with the band-pass filter shown in **b**. **(b)** Magnitude-response estimate of the band pass filter obtained by using the filter parameters shown in the panel.

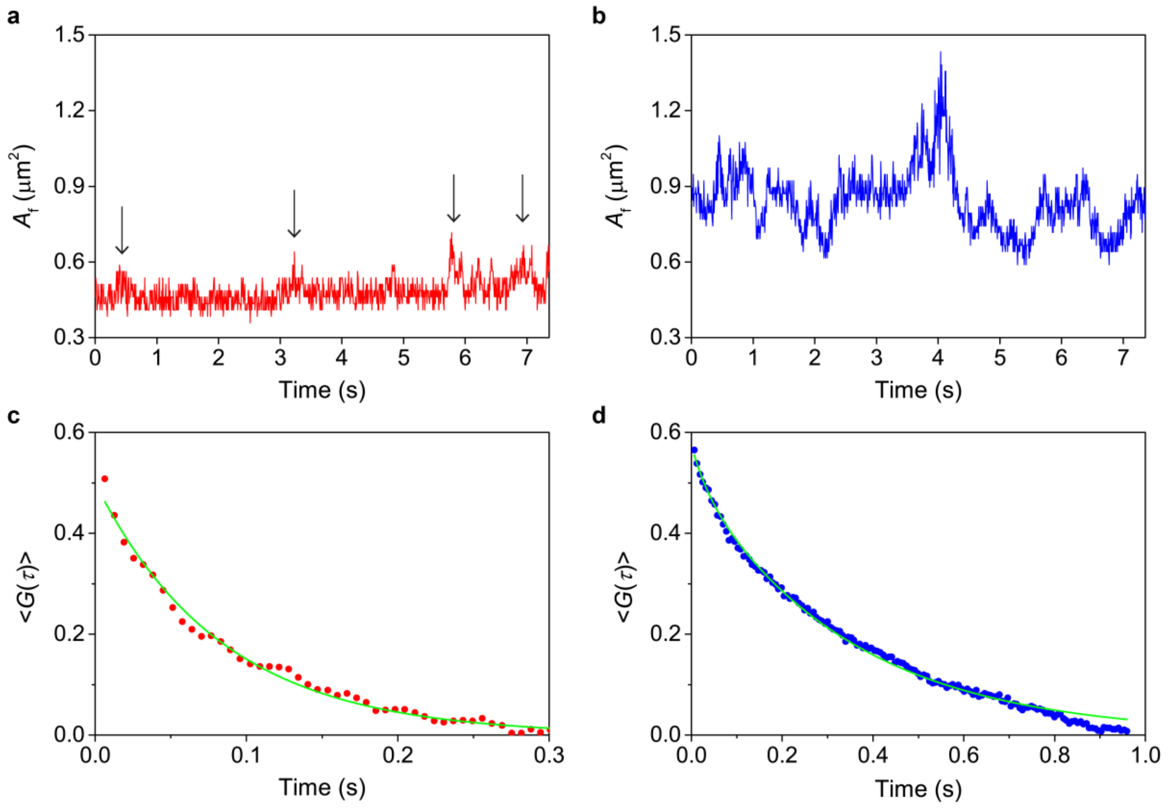

Supplementary Figure 11. Calculation of the conformational relaxation time of ColE$_1$ DNA. **(a)** Time lapse of the area occupied by a nanosphere. The slow fluctuation of the area occupied by the nanospheres (A_f) arises due to brief partial escape of the particle from the field of view (defocusing-fluctuations; black arrows). **(b)** Time lapse of the area occupied by a ColE$_1$ DNA molecule (A_f). **(c)** Averaged autocorrelation plot $\langle G(\tau)\rangle$ of the A_f of 50 nanospheres. The characteristic time of the defocusing-fluctuations (τ_n = 0.083 s) was calculated by fitting their autocorrelation function $\langle G(\tau)\rangle$ to a single-exponential decay (Eq. 16). **(d)** Averaged autocorrelation plot $\langle G(\tau)\rangle$ of A_f of 35 DNA molecules. The conformational relaxation time (τ_R = 0.34s) was calculated by using Eq. 18.

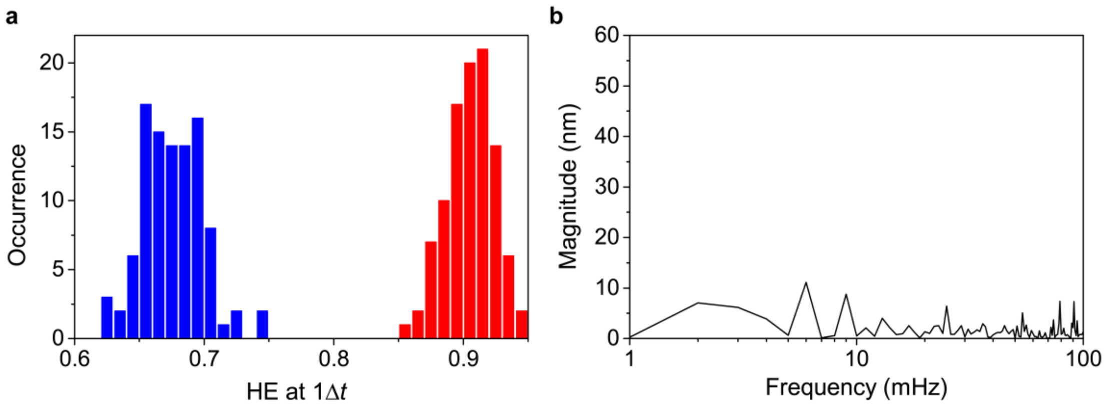

Supplementary Figure 12. Lattice occupancy analysis of ColE$_1$ and lambda DNA. (a) Frequency histogram of the Hurst exponent (HE) at 1Δ*t* of the experimental and the S_rA_r simulated replicates of ColE$_1$ DNA. **(b)** Frequency-magnitude spectrum of the c-LO extended data set of a lambda DNA experimental replicate. The spectrum shows no periodic displacement signal in the c-LO extended data set.

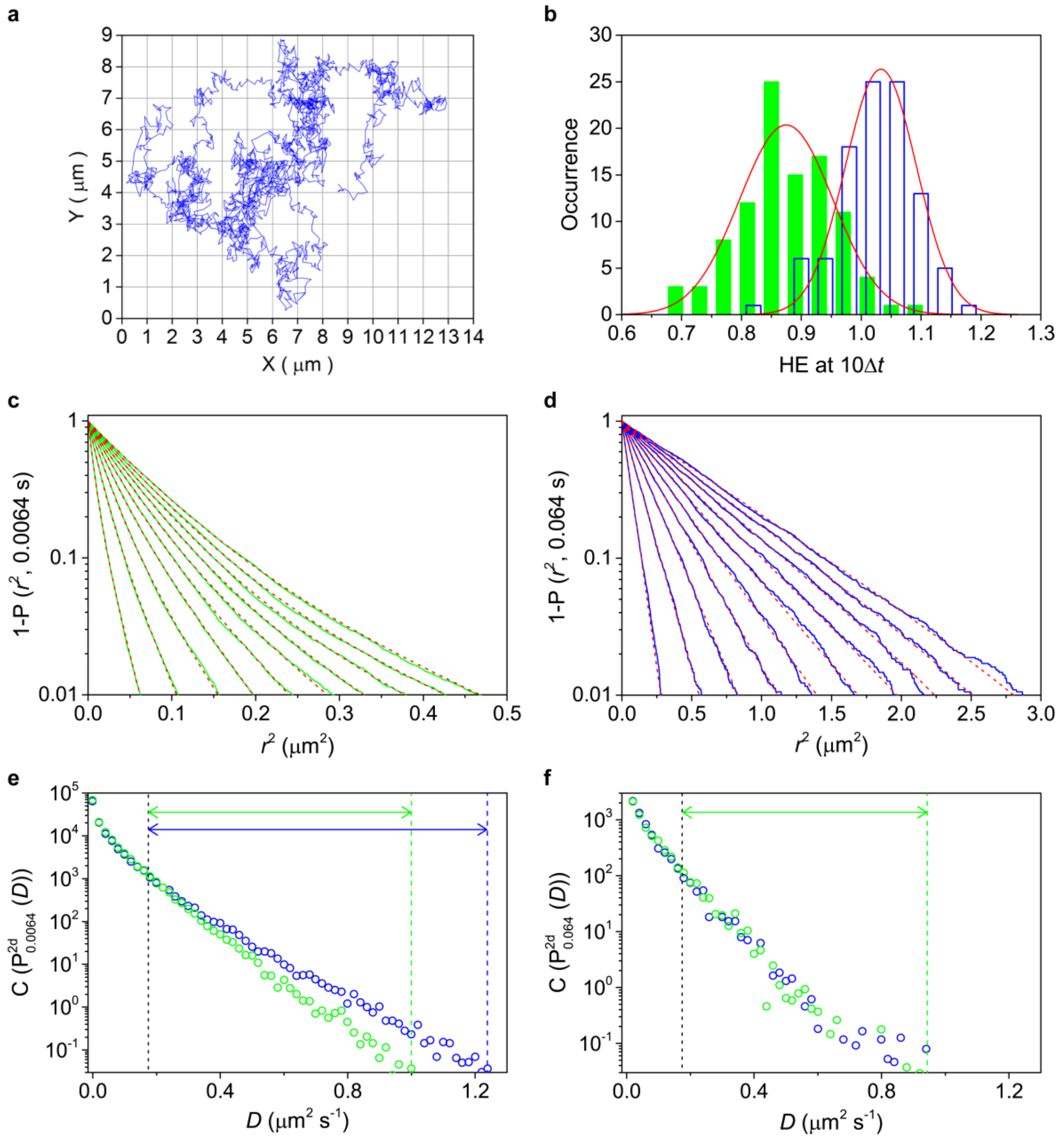

Supplementary Figure 13. Comparison of lattice occupancy analyses of molecular motion with the absolute measurements using current theories. (a) Experimental replicate of $ColE_1$ DNA probed at 10Δ*t*. **(b)** Frequency histograms showing the distributions of the calculated Hurst exponents (HE) of the experimental (blue) and the simulated S_rA_r (green) replicates probed at 10Δ*t* (the original HE profiles are shown in Fig. 3c). The solid lines are Gaussian fittings of the frequency distributions. The shift in the HE values, which we obtained by probing the motion at 10Δ*t*, confirmed the existence of non-random behaviours that have time scale(s) longer than the time required by the molecule to diffuse a distance equal to its radius of gyration (8Δ*t*; Fig. 2c). **(c)** The cumulative distribution functions (CDFs), in the form of (1–*C*) (green lines, obtained from Supplementary Equation 1), of an experimental replicate probed at 1Δ*t*. The red dashed lines show

double-exponential fittings of the CDFs. We attribute the fitting to double-exponential decay to the sub-diffusive behaviour of the DNA molecules (Fig. 2c). **(d)** Cumulative distribution functions (CDFs) (blue lines) of the experimental replicate probed at 10Δ*t*. The red dashed lines show single-exponential fittings of the CDFs and this indicates homogeneous diffusion. The CDF and the MSD analyses are sensitive to non-random behaviours that could arise from the perturbations in the distribution of the spatial positions of the molecules during a diffusion process. Therefore, based on our results in **c** and in Supplementary Fig. 3a, we conclude that CDF and MSD analyses are not sensitive to the non-random relative behaviours that we report in this study. **(e)** Distribution of generalized diffusivities (DOGD) of the time series of step sizes probed at 1Δ*t*. The DOGD algorithm describes a distribution of the fluctuations of the step sizes (not the positions; c.f. CDF and MSD analyses) around the generalized step size (*r* in Eq. 5) of the time series. The black dashed line shows the generalized diffusion constant (D_c in Supplementary Equation 3) of the DNA molecules (D_c = 0.17 μm^2 s^{-1}). We found that the width (the blue double arrow) of the experimental DOGD (the blue circles) is larger than the width (the green double arrow) of the random simulation (random distribution of step sizes obtained from Eq. 5; the green circles) and this indicates a non-ergodic time series. The ergodicity breaking in this time series occurs because of the presence of different distributions of the step sizes (Fig. 7a) that underlie the temporal profile of the step sizes obtained from the experimental replicate. **(f)** DOGD of the time series of step sizes probed at 10Δ*t*. We found that the experimental distribution (blue circles) overlaps with that of the random simulation (green circles) and that this apparently indicates an ergodic process of homogeneous isotropic diffusion.[1, 2] Because the motion in this analysis was probed at 10Δ*t*, we conclude that the DOGD analysis is not sensitive to non-random perturbations at the 10Δ*t* time scale compared with our lattice occupancy analysis, as shown in **b**. In addition, although we were able to detect the perturbations in step sizes by using the DOGD analysis at 1Δ*t* (**e**), the analysis is not designed to uncover the associated diffusion modes and the step to mode matching that we detected in Figs. 7 and 8 in the main text. Taken together, these results highlight how the relative motion of DNA is supremely sensitive to the elusive dynamics of DNA motion.

Supplementary Note 1: Single-molecule localization of $ColE_1$ DNA

A. Single-molecule localization of DNA

We used a published single-molecule localization and tracking algorithm.[3] The algorithm uses a mixture-model fitting algorithm to localize and track multiple particles in the same field of view. Furthermore, it can detect the merging and splitting of particles during motion. To achieve these tracking targets, the algorithm localizes and tracks all the local maxima in the single-molecule image including maxima that are partially overlapping. As discussed in Supplementary reference 4, the algorithm is universal and can track long molecules as well as point-like particles. We provide the below details on how we exploited this algorithm to determine the position of the 6.6 kbp molecule.

To illustrate the performance of this algorithm in our data analysis, we consider the molecule shown in Supplementary Video 1. The single-molecule image of this relatively short dsDNA contains up to 1-2 fluorescence maxima (Fig. A1). The algorithm detects and tracks both of them independently (Fig. A2). Figure A1a shows the $ColE_1$ DNA molecule where two fluorescence maxima were identified. These maxima (Fig. A1) were independently fitted to a two-dimensional (2D) Gaussian function to determine the position (dark blue and green circles in Fig. A1; more technical details on the robust fitting using the Gaussian kernel and the tracking algorithms can be found in Fig. 1, Eq. 1-6 and in supplementary notes 3 and 4 of Supplementary reference 4). These two maxima were independently tracked as shown in Fig. A2. If one of the fluorescence maxima is lost due to the escape of the molecule from the field of view (Fig. A3), its tracking is terminated while the tracking of the other maximum is continued (Fig. A4). We emphasize the following two points. First, in our analysis, we considered only one track per one molecule. We chose the track that corresponded to the brightest local maxima in the single-molecule image (i.e., track 1 in Fig. A4). Second, the reason why we used the localization and tracking algorithm and not the centre of mass calculations is presented in section B.

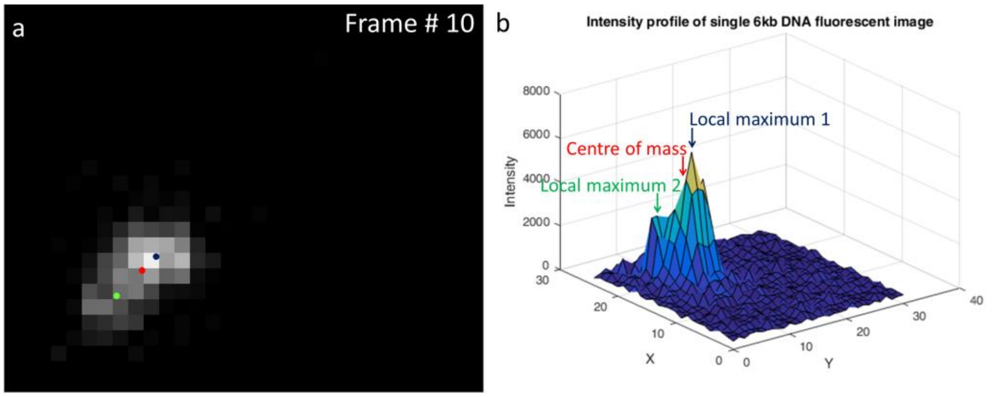

Figure A1. Intensity profiles of the DNA shown in Supplementary Video 1. The localization and tracking algorithm detected two fluorescence maxima (blue and green dots) as shown in **a**. The dark blue and green arrows in **b** indicate fluorescence maxima 1 and 2, respectively. The dark blue and green circles indicate the position after independent fitting of both maxima to the Gaussian kernel. The red arrow and red point in **a** and **b** indicate the calculated centre of the mass.

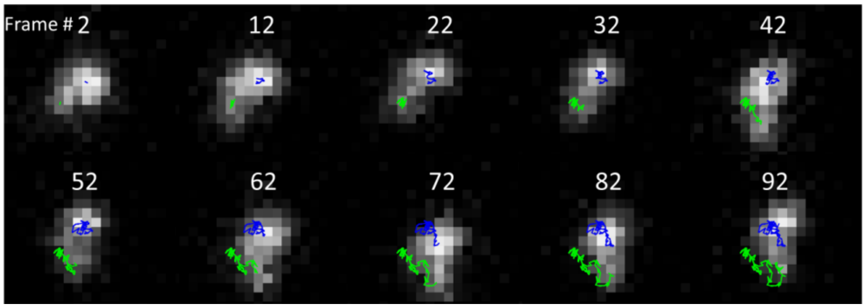

Figure A2. The molecular tracks (frame 2 ~ frame 92) of the DNA shown in Supplementary Video 1. The track number is shown for each image. The blue track is a track of fluorescence maximum 1 (see Figs. A1), whereas the green track is a track of local maximum 2.

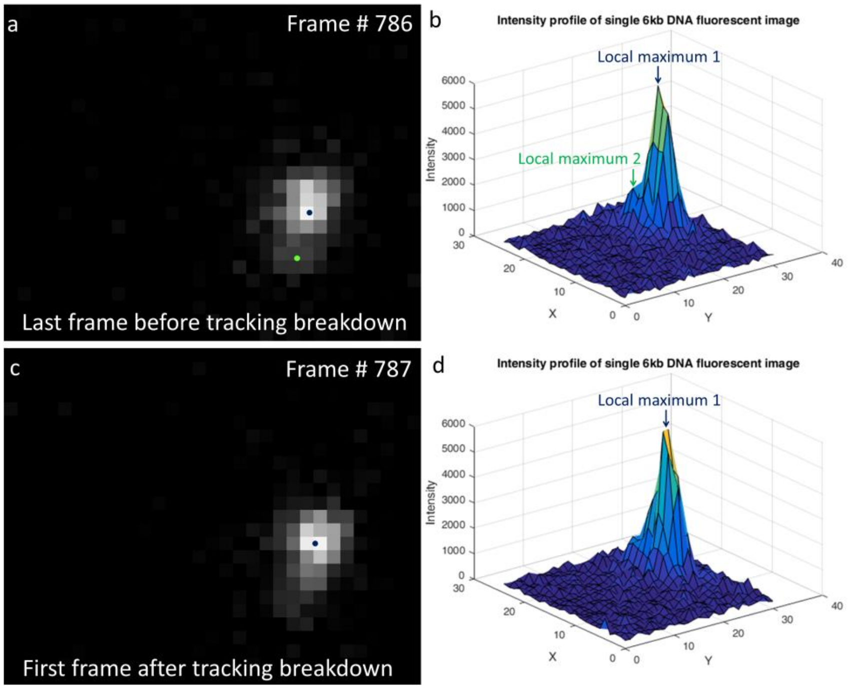

Figure A3. Intensity profiles of frame 786 (**a,b**) and frame 787 (**c,d**) of the DNA shown in Fig. 2a. The dark blue and green arrows indicate fluorescence maxima 1 and 2, respectively. The dark blue and green circles indicate the position after fitting using the Gaussian kernel of fluorescence maxima 1 and 2, respectively. The localization and the tracking algorithms detected two maxima in frame 786 but not in frame 787. In frame 787 only one maximum (1) was detected.

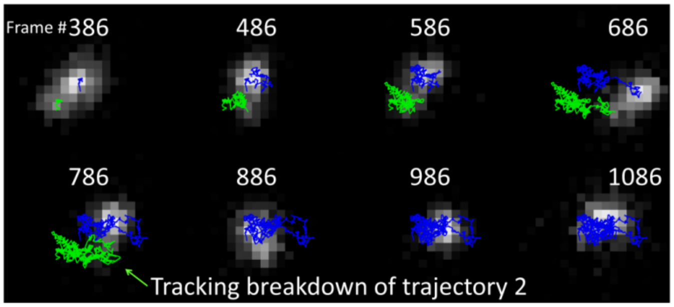

Figure A4. The molecular tracks (frame 386 ~ frame 1086) of the DNA shown in Supplementary Video 1. The track number is shown for each image. At frame 786, the tracking of trajectory 2 (green; fluorescence maximum 2) was terminated (see Figs. A3), whereas the tracking of trajectory 1 (blue; fluorescence maximum 1) is continued.

B. Single-molecule tracking in comparison with centre-of-mass calculations

We compared results based on the centre-of mass-algorithm (COMA, calculation of the centroid position) with our results based on the localization and tracking algorithm (LTA).

To highlight the similarities/differences between COMA and LTA in our analyses, we consider the 6.6 kbp molecule shown in Supplementary Video 1. The distributions of the calculated displacements by LTA and COMA are shown in Figs. B1a-c. For clarity and to ease comparison, we show only the first 300 tracks of both calculations in Figs. B1d, e. We found that the distribution of displacements obtained by LTA resulted in better fitting to normal 2D diffusion theory (Eq. 5) compared with those obtained by COMA (Fig. B1c). Although we found that the calculated displacement value (r) from the fitting was approximately the same in both calculations (Figs. B1a, b), we decided to track the molecules using the algorithm that resulted in a better fitting of the displacement data to normal 2D diffusion theory (Eq. 5, Fig. B1c). This explains why we used LTA and not COMA in our analyses.

Indeed, we checked whether we could detect the same deviations in the relative motion in the tracks obtained using COMA. To that end, we generated 100 experimental replicates and 100 S_rA_r simulated replicates from the COMA tracks. We applied the same analytical procedures as explained in the manuscript to detect and analyse the tracks of the relative motion of the DNA molecules. We found that COMA resulted in cumulative HE profiles (Fig. B1f) similar to those shown in Figure 3e in the main text of the paper (obtained from the TLA tracks). We therefore conclude that the minor differences between the LTA and the COMA tracks do not affect our results.

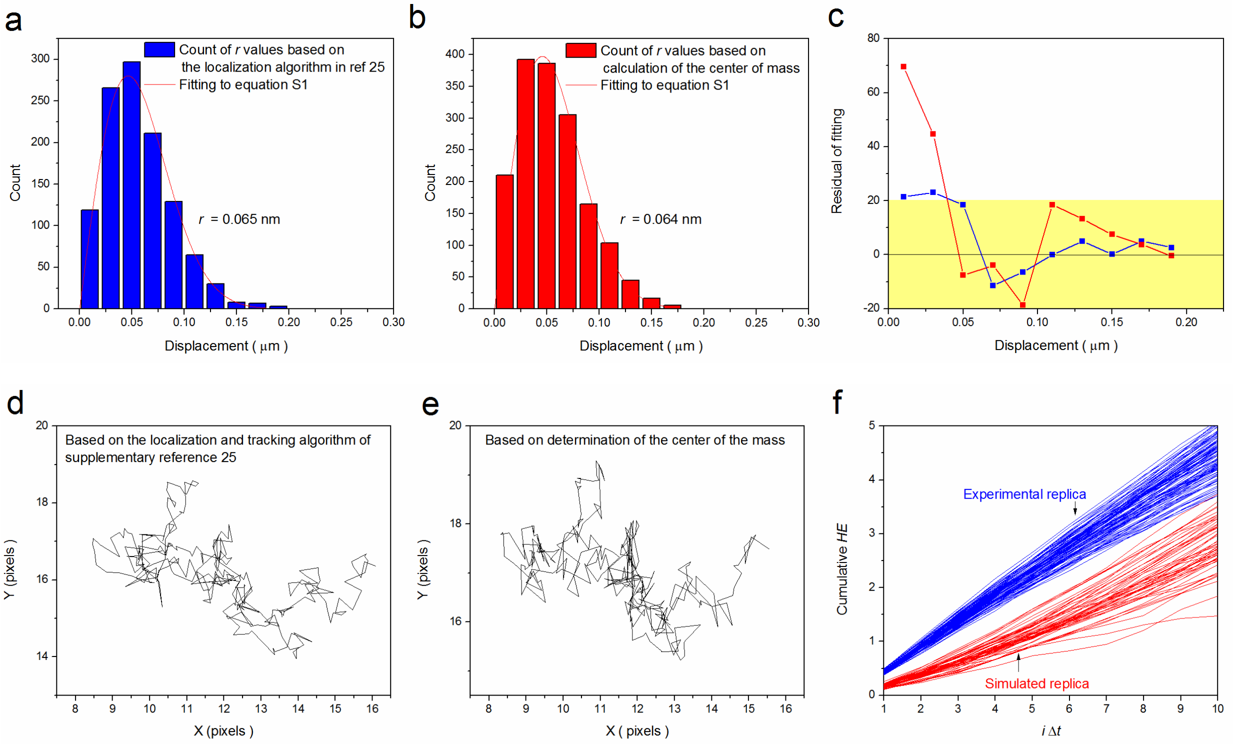

Figure B1. Comparison of the calculations based on LTA and COMA. **(a,b)** The distribution of displacements obtained by using LTA (**a**) and COMA (**b**). The calculated displacement value (*r*), after fitting the data (the red line) to equation 5, is shown for each algorithm. **(c)** The residuals of fit of the displacement data in (**a** and **b**) to Eq. 5. The blue plot shows the residuals for LTA, whereas the red plot shows the residuals for COMA. **(d,e)** Molecular tracks of the 6.6 kbp DNA obtained by using LTA (**d**) and COMA (**e**). **(f)** Cumulative Hurst exponents (HE) at different time lags ($i\Delta t$) of the relative modes of the experimental replicates (blue) and the S_rA_r (red) simulated replicates. These data were obtained from the COMA tracks. The data on the LTA tracks are shown in Fig. 3e.

Supplementary Note 2: Supplementary Software

The analyses can be done by running the file Main.m in the Supplementary software folder. In the following table we describe the input variables of the file Main.m.

Input variable	Notes
`Parameters.Cores`	Number of the processor cores to be used in the probability calculations and in the dynamic time-warping analysis. This number depends on the specifications of the machine. The default value is 4 cores.
`Parameters.File_name`	The name of the file that contains the coordinates of the joined trajectories. In this software, the lattice size is equal to the pixel size. The unit of the coordinates should therefore be in pixels. The data should be only numbers and in two (X and Y) columns.
`Parameters.Trajectory_length`	The length of the trajectory to be analyzed. The length of the joined trajectories should be long enough to allow robust calculations of HE. The default value is 29360 time frames.
`Parameters.Pixel_size`	The pixel size of the camera in micrometers. The default value is 0.16 μm.
`Parameters.Frame_rate`	The frame rate in seconds. The default value is 0.0064 seconds.
`Parameters.Sliding_window_size`	The size of the sliding window that is used in the calculations of the time-dependent probability and the time-dependent MSD. See Methods. The unit is in frames. The default value is 50 frames.
`Parameters.PLE_limit.A=50`<br>`Parameters.PLE_limit.B=5000`	The size limits of the detrending segments of the detrended fluctuation analysis (DFA). See Methods. The unit is in frames. The default values are 50 and 5000 frames.
`Parameters.Confined_limit`	The fitting limit of the side length of the confined area in micrometers. This parameter is empirically obtained from the MSD curve. Details are included in Methods. The default value of $ColE_1$ DNA at our experimental conditions is 700 nm. This value should be independently calculated for each experimental and imaging condition.
`Parameters.DFA_order`	The fitting order in the detrended fluctuation analysis (DFA). The default value is 1. This value should be the same when comparing the experimental and simulated trajectories.

`Parameters.DTW_window_size`	The size of the sliding window of the dynamic time warping (DTW) analysis. The unit is in frames. The default value is 50 frames.
`Parameters.DTW_Mstd`<br>`Grand Mean - (1.28 * Standard deviation of the time-warped signal)`	This parameter is used to specify the amplitude threshold that we use in the third step of our four-step analytical. The default value of the grand mean ($ALV\mu_G$) is -0.5747 and the default value of z is 1.28σ. The default value of σ is 3.742.

The software contains the detailed calculations for the detection and the analysis of the CLO sub-mode. Other sub-modes can be analyzed by changing the relevant parameters. To analyze the DHO sub-mode instead of the CLO sub-mode, the following steps should be followed:

1- `Parameters.DTW_Mstd` should be changed to Mean + (1.28 * Standard deviation of the time-warped signal).
2- The first mode-set (see Fig. 5) has to be analysed by changing the logical sign (<), in lines 45 and 47 in the Main2.m, to the logical sign (>).
3- Line 16 in the Disp_resolve.m file should be deleted.
4- "`DTW_TS_INV`" in Line 17 of the Disp_resolve.m file should be replaced by "`DTW_TS`".

In the following table, we present benchmark results of the analysis. The software has been tested on Matlab versions 2014b, 2015a and 2016a

Calculation platform	Matlab version	Analysis time	Notes/number of cores
Intel 2× Xeon CPU E5-2687W v2 @ 3.40GHz Windows 8 (64 bit)	2016a	12 minutes	16 cores
Intel 2× Xeon CPU E5-2687W v2 @ 3.40GHz Windows 8 (64 bit)	2014b	15 minutes	16 cores
Intel Xeon CPU X-5650 @ 2.67GHz Windows 7 (64 bit)	2012a (This Matlab version lacks the necessary functions to do the band-pass filtering of the periodic signal. Also it needs a different command to run the parfor loops.)	15 minutes (All analyses except the band-pass filtering)	12 cores
Intel Core i7-3770 CPU @ 3.40GHz Windows 8 (64 bit)	2015a	43 minutes	4 cores

The following table shows the output variables of the supplementary software.

Output variable	Description
Coordinates_M	This variable contains the coordinates of the joined trajectories. The trajectory can be visualized by running `plot (Coordinates_M(:,1),Coordinates_M(:,2))`
Prob_TS	This variable contains the probability time series. The time series can be visualized by running `plot ([1:length(Prob_TS)]*Parameters.Frame_rate,Prob_TS)`
MSD_TS	This variable contains the MSD time series. The time series can be visualized by running `plot ([1:length(MSD_TS)]*Parameters.Frame_rate,MSD_TS)`
ProbabilityNorm & MSDNorm	These variables contain the inverted second mode-set of the normalized probability and the normalized MSD time series respectively. Both time series can be visualized by running `plot ([1:length(ProbabilityNorm)]*Parameters.Frame_rate, ProbabilityNorm)` `hold on` `plot ([1:length(MSDNorm)]*Parameters.Frame_rate,MSDNorm)`
Hurst_Probability	Hurst exponent of the probability time series
Displacement_CLO	The joined displacement time series of the CLO sub-mode. The time series can be visualized by running `plot (Displacement_CLO)` Refer to Supplementary Fig. 9 for details
Displacement_CLO_filtered_Normalized	This variable contains the filtered signal of the CLO sub-mode. Refer to Supplementary Fig. 9 for details. The filtered signal can be visualized superimposed on the original signal by running `hFig = figure(1);` `A=get(0,'MonitorPositions');` `Y_pos=A(1,4)-(A(1,4)/3)-100;` `set(hFig, 'Position', [1 Y_pos A(1,3) A(1,4)/3])` `plot (Displacement_CLO_filtered_Normalized);` `hold on; plot (Displacement_CLO)`
F & pxx	The output variables of the Fourier-transform. These variables can be visualized by running `plot (1000*f/length(Displacement_CLO),pxx)`

Supplementary Note 3: Rational for splitting the P_{25} and MSD profiles with respect to μ_{nP}.

We split the two time series to independently analyse each absolute mode (i.e., directed- or confined-like motion) with the appropriate relative mode (low or high occupancy). This splitting allows sub-modes of motion that might emerge in each mode-set to be more easily distinguished. In the first mode-set, the directed-like absolute mode whose normalized values are greater than μ_{nP} was analysed with the low lattice occupancy mode ($P_{25} > \mu_{nP}$). In the second mode-set, the confined-like absolute mode whose normalized values are less than μ_{nP} was analysed with the high lattice occupancy mode ($P_{25} < \mu_{nP}$).

We split the two time series to distinguish between different dynamics that could show similar ALV values. For example, in Fig. 5 Step A of the main text, if the two time-series are analysed without the splitting, the ALV values calculated from peaks 7 and 12 would be similar to the ALV values calculated from peaks 3 and 10. The analysis without splitting could not thus discern which part of the time series (peak 7 or peak 3) had higher normalized amplitude. On the other hand, the splitting of the two time series generates ALV values relative to the μ_{nP} value and thus the splitting could help to identify that the amplitude of peak 7 is larger than peak 3 (Fig. 5 Step B1 of the main text). This piece of information is important to characterizing the fluctuations of the two time series in a comprehensive way and to accurately resolve the non-random modes that underlie the motion of DNA.

In a given mode-set, we lose information on the actual normalized amplitudes of the corresponding modes of the other mode-set by following this splitting (*e.g.*, we lose information on peak 12 while analysing peak 7 in Fig. 5 Step A of the main text). However, we later correct this and recharacterize those modes by using MSD and step-size distribution analyses (Fig. 5 Step D, Figs. 8a, 8b).

Supplementary Methods

Cumulative distribution function analysis

We conducted the cumulative distribution function (CDF) analysis using a routine written in Matlab. CDF is the cumulative probability, $C\ (r^2, i\Delta t)$, of finding the molecule within radius r from the origin at time lag $i\Delta t$:

$$1 - C(r^2, i\Delta t) = 1 - \int_0^r c(r^2, i\Delta t)\mathrm{d}r = \exp\left[-\frac{r^2}{4D(i\Delta t)}\right]. \qquad \text{(Supplementary Equation 1)}$$

This algorithm disregards the individuality of the trajectories to analyse the distribution of all displacements from the combined trajectories at each time lag.[4]

Distribution of generalized diffusivities analysis

We conducted the distribution of generalized diffusivities (DOGD) analysis using a routine written in Matlab. DOGD relates each displacement (x) of a single-molecule trajectory in U dimension(s) to the so-called single-molecule diffusivity, D_t, which is given by[2]

$$D_\mathrm{t}(\tau) = \frac{[x(t+\tau)-x(t)]^2}{2U\tau}\ . \qquad \text{(Supplementary Equation 2)}$$

The distribution of generalized diffusivities (V) (equation S3) fluctuates around the generalized diffusion coefficient (D_c) and is given by[2]

$$V_{D_\mathrm{c}}^{Ud} = \left(\frac{U}{2D_\mathrm{c}}\right)^{\frac{U}{2}} \frac{D_\mathrm{t}^{\frac{U}{2}-1}}{\Gamma(U/2)} \exp\left(-\frac{U}{2D_\mathrm{c}} D_\mathrm{t}\right). \qquad \text{(Supplementary Equation 3)}$$

DOGD provides a detailed analysis of the fluctuations of the local diffusion constant (D_t) around the generalized diffusion constant (D_c) during the diffusion process. Thus, DOGD analysis is regarded as a robust test for ergodicity breaking in a non-random walk.[1]

Supplementary References

1. Albers, T. & Radons, G. Subdiffusive continuous time random walks and weak ergodicity breaking analyzed with the distribution of generalized diffusivities. *Epl* **102**, 6 (2013).

2. Heidernatsch, M., Bauer, M. & Radons, G. Characterizing N-dimensional anisotropic Brownian motion by the distribution of diffusivities. *J. Chem. Phys.* **139**, 14 (2013).

3. Jaqaman, K. *et al.* Robust single-particle tracking in live-cell time-lapse sequences. *Nat. Methods* **5**, 695-702 (2008).

4. Habuchi, S., Satoh, N., Yamamoto, T., Tezuka, Y. & Vacha, M. Multimode Diffusion of Ring Polymer Molecules Revealed by a Single-Molecule Study. *Angew. Chem. Int. Ed.* **49**, 1418-1421 (2010).
