# Supplementary figures and images for: Conserved linear dynamics of single-molecule Brownian motion
Source: Nat Commun. 2017 Jun 6;8:15675. doi: 10.1038/ncomms15675 (PMC5467176; doi:10.1038/ncomms15675)

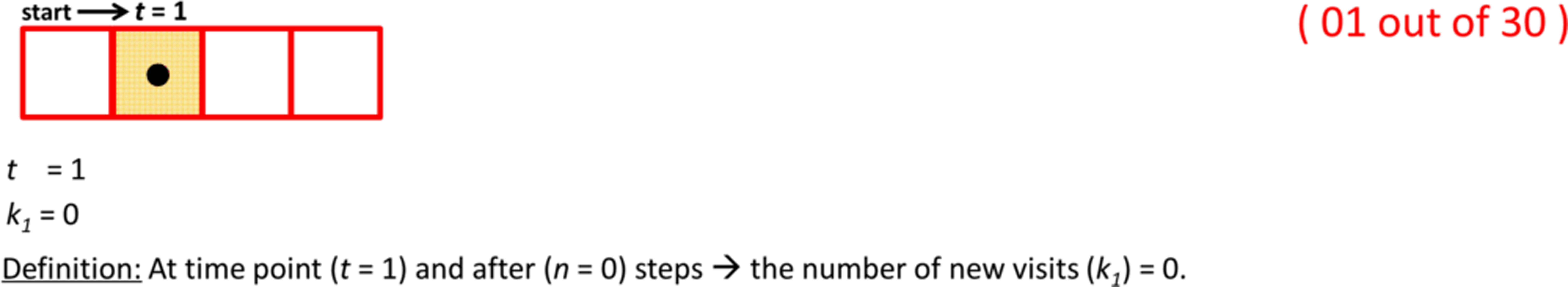

Supplement: Supplementary Data 1 — Step-by-step description of lattice occupancy analysis. Equations 1 and 3 are derived in this scheme. [file ncomms15675-s4.tif]

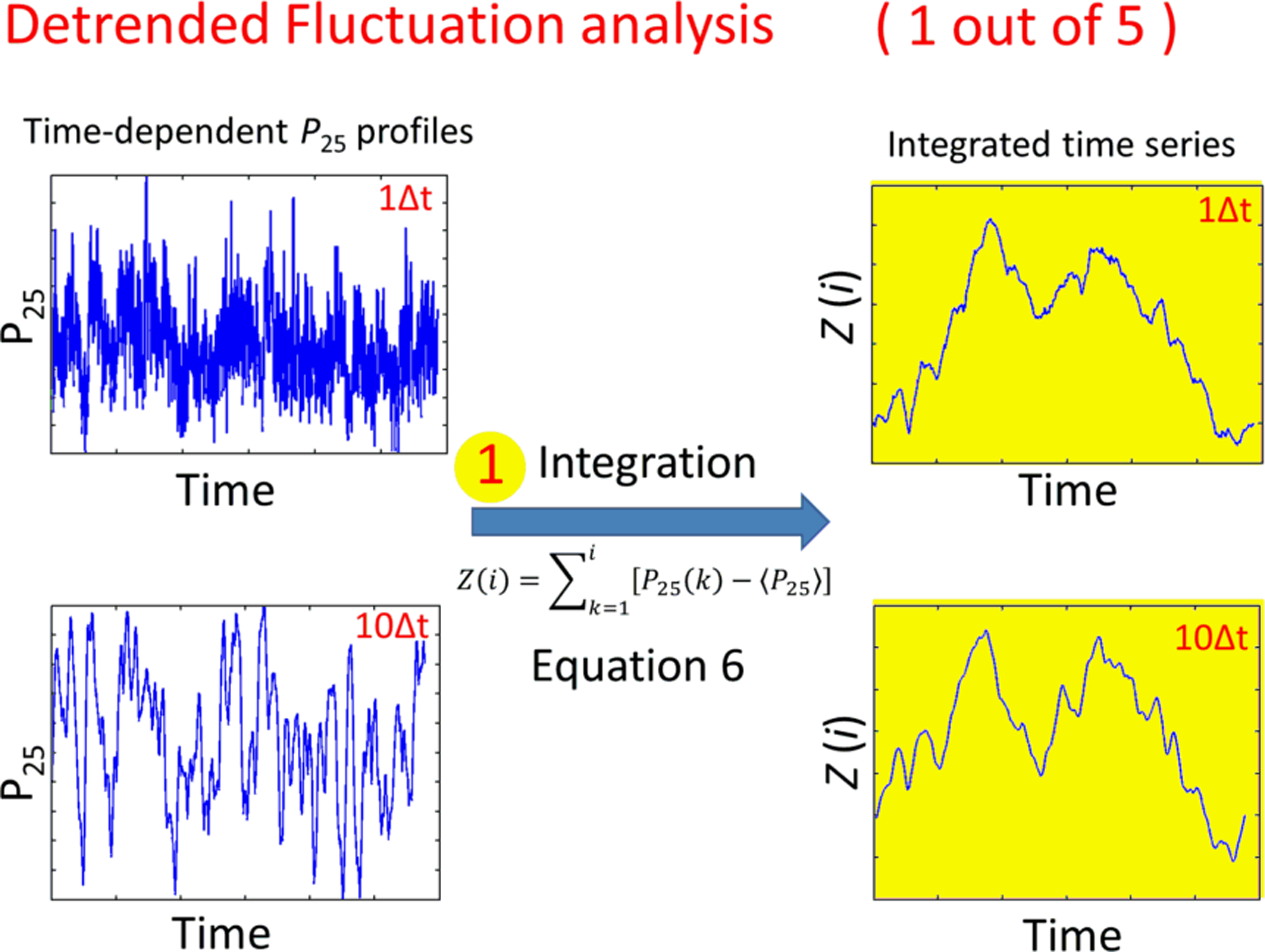

Supplement: Supplementary Data 2 — Step-by-step description of detrended fluctuation analysis. The calculations of the Hurst exponents (HE) from the temporal profiles of P25 using equations 6, 7, and 8 are described in detail. [file ncomms15675-s5.tif]

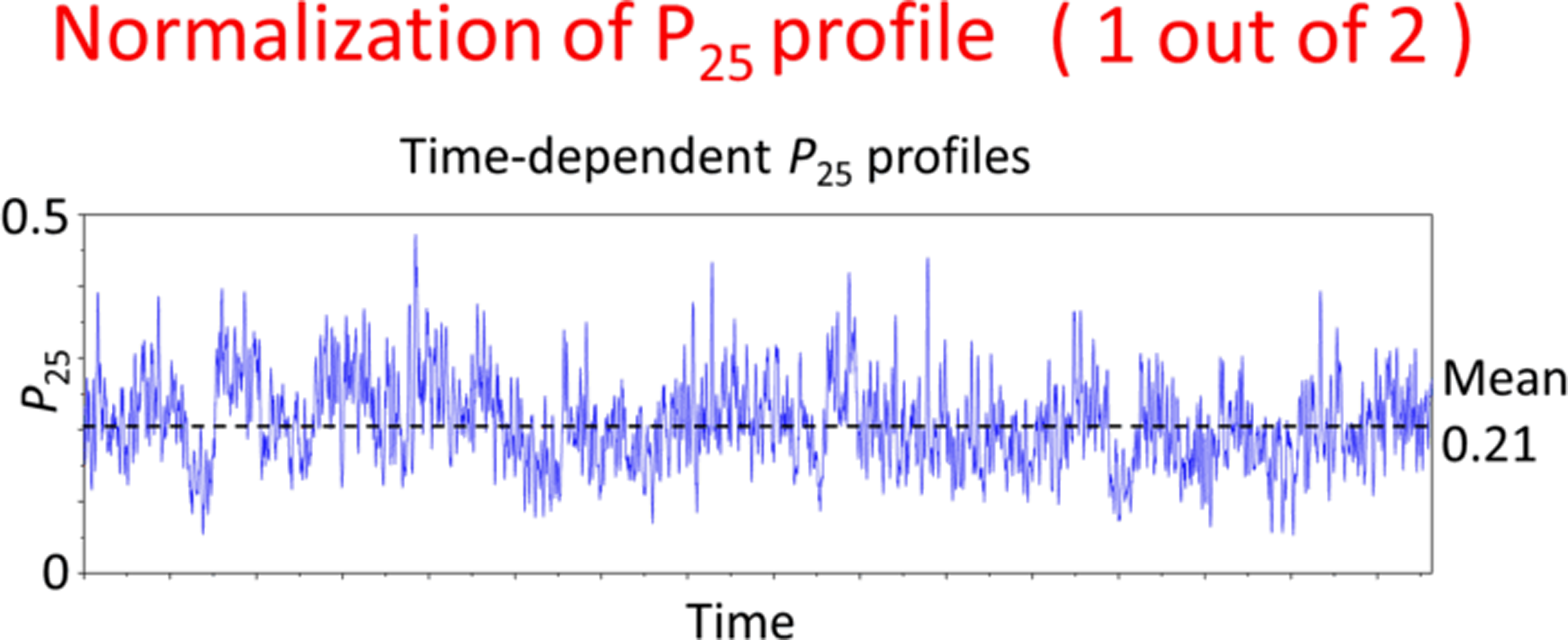

Supplement: Supplementary Data 3 — Normalization of P25 temporal profile. [file ncomms15675-s6.tif]

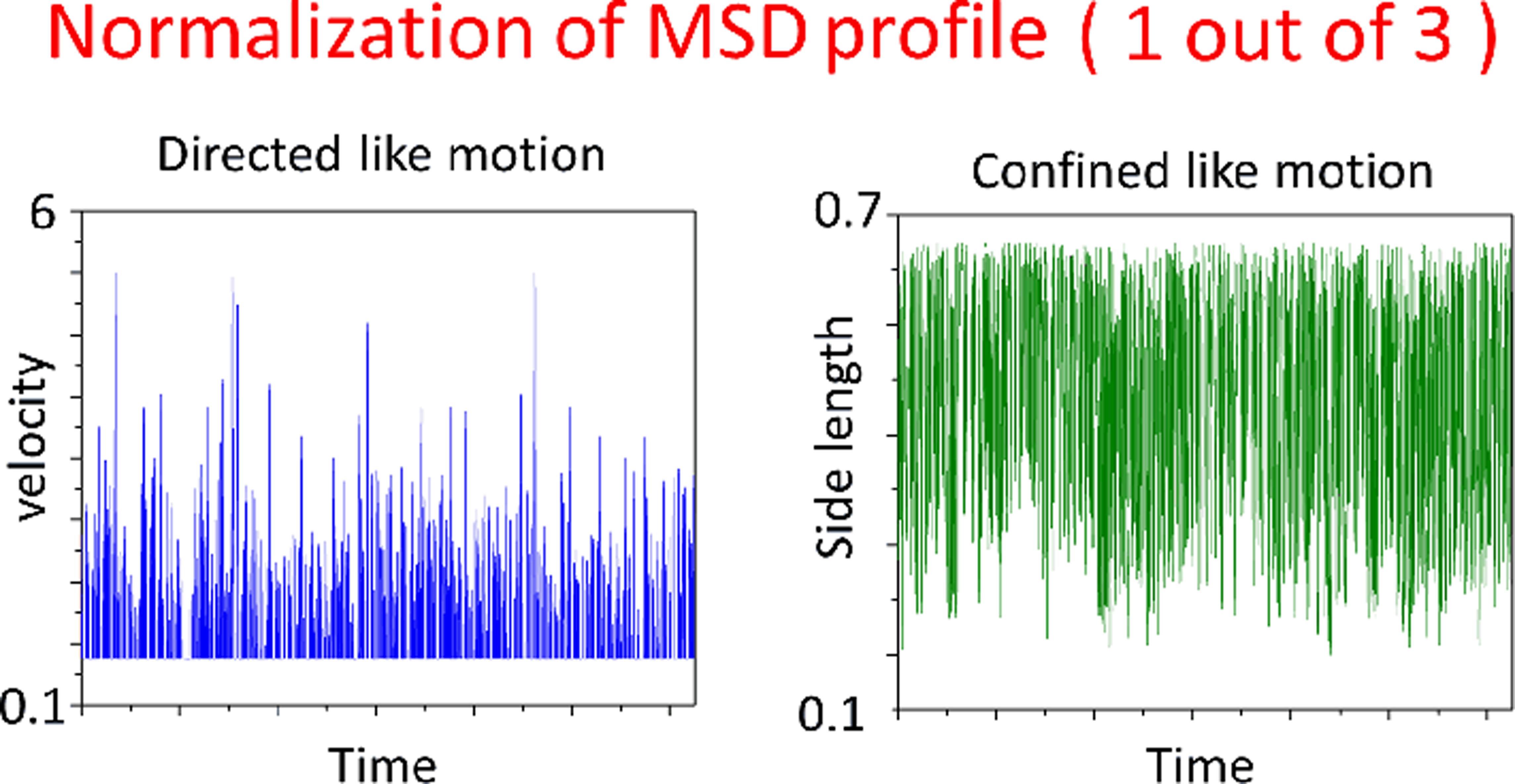

Supplement: Supplementary Data 4 — Normalization of temporal MSD profile. [file ncomms15675-s7.tif]

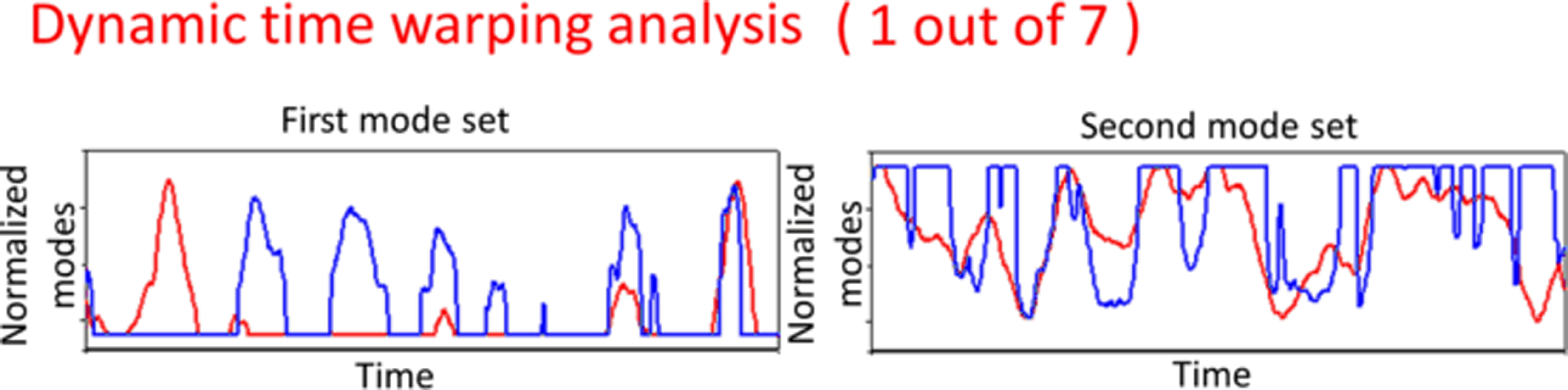

Supplement: Supplementary Data 5 — Step-by-step description of dynamic time warping analysis. The calculations of the amplitude of local variations (ALV) from the temporal profiles of P25 and temporal MSD profiles using equations 12, 14, and 15 are described in detail. [file ncomms15675-s8.tif]

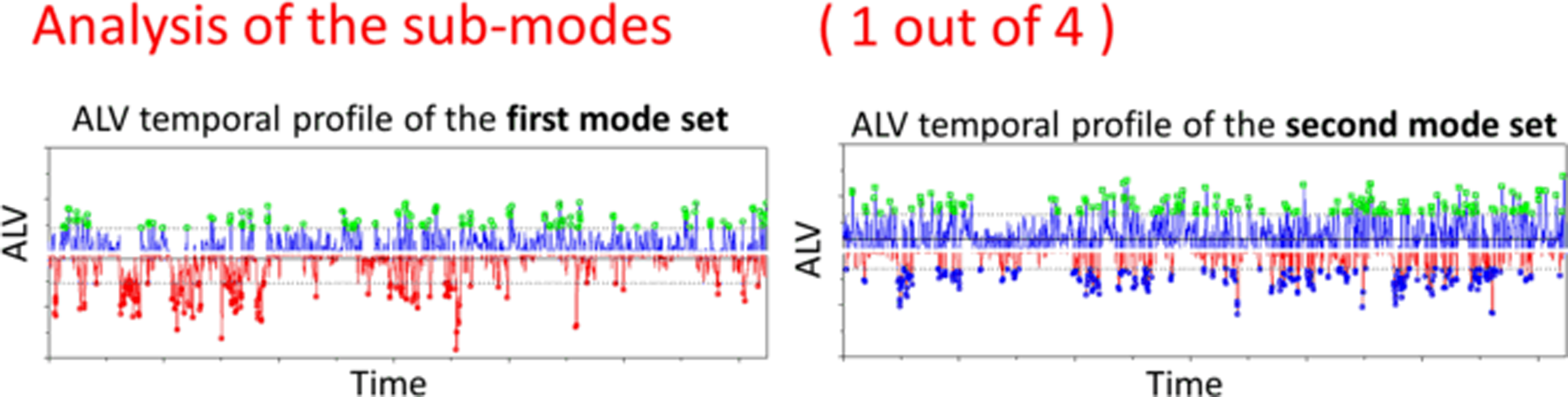

Supplement: Supplementary Data 6 — Step-by-step description of the analysis of the sub-modes. The procedures of determining the step-sizes and MSD-?t profiles of each sub-mode are described in detail. [file ncomms15675-s9.tif]

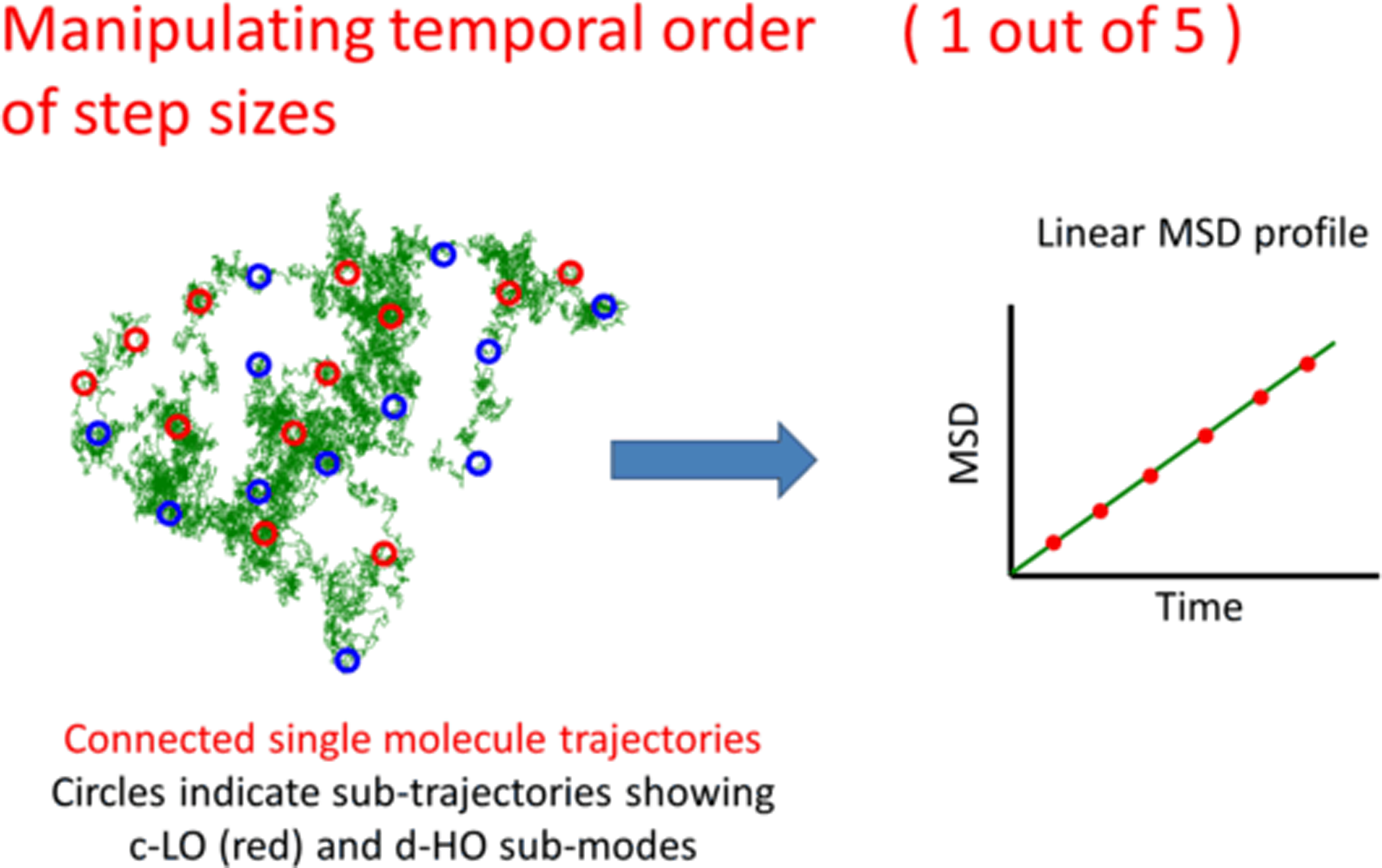

Supplement: Supplementary Data 7 — Step-by-step description of the manipulation of the temporal order of the step-sizes. The procedures of calculating the MSD-?t profiles of the manipulated single-molecule trajectories, in which the step-sizes of c-LO or d-HO sub-modes in the original trajectories are replaced by those of other sub-modes, are described in detail. [file ncomms15675-s10.tif]
